# Commentary: Epidemiology of Antibody-Positive Autoimmune Encephalitis in Southwest China: A Multicenter Study

**DOI:** 10.3389/fimmu.2020.01976

**Published:** 2020-08-28

**Authors:** Yanbing Zhou, Zirui Meng, Binwu Ying

**Affiliations:** Department of Laboratory Medicine, West China Hospital, Sichuan University, Chengdu, China

**Keywords:** autoimmune encephalitis, epidemiology, neuronal autoantibodies, thyroid peroxidase antibodies, glasgow outcome scale, modified rankin scale, response to immunotherapy in epilepsy and encephalopathy score

Recently, Gu et al. reported an epidemiological survey about autoimmune encephalitis (AE) in southwestern China, involving six large general hospitals in Chongqing (1). Their study revealed the underlying relationship between several factors and disease severity (1). Although there have been no large-scale epidemiological investigations of AE in China prior to this report (1), the conclusions of this research would have been more reliable had the following concerns been addressed.

First, the limited scale of this epidemiological investigation merits discussion. The detailed epidemiological features of AE in southwestern China were presented in this multicenter study involving six large general hospitals in the Chongqing area. These six general hospitals in Chongqing were limited to reflect the epidemiological features of AE patients in the southwestern region of China.

Moreover, it is noteworthy that researchers excluded patients with thyroid disease (1). However, some patients with Hashimoto's encephalopathy (HE), which is an important cause of autoimmune encephalopathy, may be neglected. High titers of thyroid peroxidase antibodies (TPO-Ab) is generally detected in HE patients (2). And morbidity of HE is estimated to be 2.1/100,000 in adults (3). Additionally, TPO-Ab detection was recommended to be tested in the systematic diagnosis per the clinical diagnosis criteria of AE published in 2016 (4). Therefore, it is quite likely to miss potential patients with AE by excluding patients with thyroid disease.

Also, the methods of antibody detection in the publication need to be further elaborated. According to the 2016 clinical diagnosis criteria of AE, antibody detection in definite AE-like encephalitis with anti-NMDA receptor antibody-positive status should include cerebrospinal fluid (CSF) testing with cell-based assay (CBA) and with confirmatory tests like tissue immunohistochemistry based on animals' brain tissue (4). The tissue immunohistochemistry has been widely used by some studies of AE (5–7). In the author's study, only CBA based on indirect immunofluorescence (IIF) assay was performed to analyze both the CSF and serum of each patient (1). Thus, owing to significant inter-operator variability in CBA performed by different technologists, the standard of determining antibody titers should be explained in detail. Further, the assessment scale of disease severity in research needs more discussion. The authors have used the Glasgow Outcome Scale (GOS) to evaluate factors that may be associated with disease prognosis (1). The GOS was initially designed to predict the outcome after brain injury-like traumas (8–11). However, some researchers have indicated that GOS has some deficiencies because it cannot detect minor brain damage (12). Therefore, we suggest that it would be better if the researchers could combine GOS with some scales that are more appropriate to predict the outcome of AE. Although no specific scale has been designed yet to predict AE prognosis (13), some studies have supported the use of the modified Rankin Scale (mRS), which is more frequently used in the evaluation of AE prognosis (14–17). Another scale called the Response to Immunotherapy in Epilepsy and Encephalopathy score (RITE2 score) has also been used to evaluate and manage autoimmune-epilepsy (18, 19).

In summary, this is a meaningful report providing epidemiological data about the antibody distribution in AE in the Chongqing area, along with revealing the factors associated with poor prognosis of AE. Despite some of the above-mentioned concerns, this study would be helpful to researchers and clinicians alike to gain more insight into AE.

## Author Contributions

All authors listed have made a substantial, direct and intellectual contribution to the work, and approved it for publication.

## Conflict of Interest

The authors declare that the research was conducted in the absence of any commercial or financial relationships that could be construed as a potential conflict of interest.

## References

[1] Gu Y Zhong M He L Li W Huang Y Liu J. Epidemiology of antibody-positive autoimmune encephalitis in Southwest China: a multicenter study. Front Immunol. (2019) 10:2611. 10.3389/fimmu.2019.02611 31781111 PMC6861323

[2] Li J Li F Hashimoto's encephalopathy and seizure disorders. Front Neurol. (2019) 10:440 10.3389/fneur.2019.00440 31133960 PMC6517482

[3] Ferracci F Moretto G Candeago RM Cimini N Conte F Gentile M. Antithyroid antibodies in the CSF: their role in the pathogenesis of Hashimoto's encephalopathy. Neurology. (2003) 60:712–4. 10.1212/01.WNL.0000048660.71390.C6 12601119

[4] Graus F Titulaer MJ Balu R Benseler S Bien CG Cellucci T. A clinical approach to diagnosis of autoimmune encephalitis. Lancet Neurol. (2016) 15:391–404. 10.1016/S1474-4422(15)00401-9 26906964 PMC5066574

[5] Ricken G Schwaiger C De Simoni D Pichler V Lang J Glatter S. Detection methods for autoantibodies in suspected autoimmune encephalitis. Front Neurol. (2018) 9:841. 10.3389/fneur.2018.00841 30364136 PMC6191500

[6] Liu T Chen B Yang H Huang J Liu S Yang X. Screening for autoantibodies in inflammatory neurological syndrome using fluorescence pattern in a tissue-based assay: cerebrospinal fluid findings from 793 patients. Multiple Scler Relat Disord. (2019) 28:177–83. 10.1016/j.msard.2018.12.036 30611927

[7] Bien CI Nehls F Kollmar R Weis M Steinke W Woermann F. Identification of adenylate kinase 5 antibodies during routine diagnostics in a tissue-based assay: Three new cases and a review of the literature. J Neuroimmunol. (2019) 334:576975. 10.1016/j.jneuroim.2019.576975 31177032

[8] McMillan T Wilson L Ponsford J Levin H Teasdale G Bond M. The Glasgow Outcome Scale - 40 years of application and refinement. Nat Rev Neurol. (2016) 12:477–85. 10.1038/nrneurol.2016.89 27418377

[9] Jennett B Teasdale G Galbraith S Pickard J Grant H Braakman R. Severe head injuries in three countries. J Neurol Neurosurg Psychiatry. (1977) 40:291–8. 10.1136/jnnp.40.3.291 886355 PMC492665

[10] Bates D Caronna JJ Cartlidge NE Knill-Jones RP Levy DE Shaw DA. A prospective study of nontraumatic coma: methods and results in 310 patients. Ann Neurol. (1977) 2:211–20. 10.1002/ana.410020306 617566

[11] Langfitt TW. Measuring the outcome from head injuries. J Neurosurg. (1978) 48:673–8. 10.3171/jns.1978.48.5.0673 641547

[12] Beers SR Wisniewski SR Garcia-Filion P Tian Y Hahner T Berger RP. Validity of a pediatric version of the Glasgow Outcome Scale-Extended. J Neurotrauma. (2012) 29:1126–39. 10.1089/neu.2011.2272 22220819 PMC3325553

[13] Lim J-A Lee S-T Moon J Jun J-S Kim T-J Shin Y-W. Development of the clinical assessment scale in autoimmune encephalitis. Ann Neurol. (2019) 85:352–8. 10.1002/ana.25421 30675918

[14] Huang X Fan C Wu J Ye J Zhan S Song H. Clinical analysis on anti-N-methyl-D-aspartate receptor encephalitis cases: Chinese experience. Int J Clin Exp Med. (2015) 8:18927–35. Available online at: http://europepmc.org/article/PMC/4694417 26770517 PMC4694417

[15] Gresa-Arribas N Titulaer MJ Torrents A Aguilar E McCracken L Leypoldt F. Antibody titres at diagnosis and during follow-up of anti-NMDA receptor encephalitis: a retrospective study. Lancet Neurol. (2014) 13:167–77. 10.1016/S1474-4422(13)70282-5 24360484 PMC4006368

[16] Titulaer MJ McCracken L Gabilondo I Armangué T Glaser C Iizuka T. Treatment and prognostic factors for long-term outcome in patients with anti-NMDA receptor encephalitis: an observational cohort study. Lancet Neurol. (2013) 12:157–65. 10.1016/S1474-4422(12)70310-1 23290630 PMC3563251

[17] Lee W-J Lee S-T Byun J-I Sunwoo J-S Kim T-J Lim J-A. Rituximab treatment for autoimmune limbic encephalitis in an institutional cohort. Neurology. (2016) 86:1683–91. 10.1212/WNL.0000000000002635 27037228

[18] Husari KS Dubey D. Autoimmune epilepsy. Neurotherapeutics. (2019) 16:685–702. 10.1007/s13311-019-00750-3 31240596 PMC6694338

[19] Dubey D Kothapalli N McKeon A Flanagan EP Lennon VA Klein CJ. Predictors of neural-specific autoantibodies and immunotherapy response in patients with cognitive dysfunction. J Neuroimmunol. (2018) 323:62–72. 10.1016/j.jneuroim.2018.07.009 30196836

